# Rates of CTL Killing in Persistent Viral Infection In Vivo

**DOI:** 10.1371/journal.pcbi.1003534

**Published:** 2014-04-03

**Authors:** Marjet Elemans, Arnaud Florins, Luc Willems, Becca Asquith

**Affiliations:** 1 Section of Immunology, Imperial College London, London, United Kingdom; 2 Molecular and Cellular Biology, Université de Liège and GIGA, Gembloux Agro-Bio Tech, Liège, Belgium; Emory University, United States of America

## Abstract

The CD8^+^ cytotoxic T lymphocyte (CTL) response is an important defence against viral invasion. Although CTL-mediated cytotoxicity has been widely studied for many years, the rate at which virus-infected cells are killed in vivo by the CTL response is poorly understood. To date the rate of CTL killing in vivo has been estimated for three virus infections but the estimates differ considerably, and killing of HIV-1-infected cells was unexpectedly low. This raises questions about the typical anti-viral capability of CTL and whether CTL killing is abnormally low in HIV-1. We estimated the rate of killing of infected cells by CD8^+^ T cells in two distinct persistent virus infections: sheep infected with Bovine Leukemia Virus (BLV) and humans infected with Human T Lymphotropic Virus type 1 (HTLV-1) which together with existing data allows us to study a total of five viruses in parallel. Although both BLV and HTLV-1 infection are characterised by large expansions of chronically activated CTL with immediate effector function ex vivo and no evidence of overt immune suppression, our estimates are at the lower end of the reported range. This enables us to put current estimates into perspective and shows that CTL killing of HIV-infected cells may not be atypically low. The estimates at the higher end of the range are obtained in more manipulated systems and may thus represent the potential rather than the realised CTL efficiency.

## Author Summary

Virus replication is countered by a range of innate and adaptive host defences. One important and widely studied adaptive defence is the CD8^+^ cytotoxic T lymphocyte (CTL) response. Quantification of the in vivo lytic capability of CTLs is essential for a detailed understanding of the immune response. This includes understanding the balance between viral replication and viral clearance, understanding the rate limiting steps in CTL killing and thus how killing can be increased and understanding the failure of CTL vaccines. However, the typical rate at which virus-infected cells are killed by the CTL response in vivo is poorly understood. Current estimates differ considerably and are especially low for HIV-1-infection. We estimated the rate of killing of infected cells by CD8^+^ T cells in two distinct persistent virus infections which enables us to put current estimates into perspective. We show that CTL killing of HIV-infected cells may not be atypically low. The estimates at the higher end of the range are obtained in more manipulated systems and may thus represent the potential rather than the realised CTL efficiency.

## Introduction

Virus replication is countered by a range of innate and adaptive host defences. One important adaptive defence is the CD8^+^ cytotoxic T lymphocyte (CTL) response which controls infection by a number of mechanisms including perforin/granzyme and Fas/FasL-mediated lysis and secretion of anti-viral cytokines. Although CTL-mediated cytotoxicity has been widely studied for many years, the typical rate at which virus-infected cells are killed by the CTL response in vivo is poorly understood as only three viral systems have been studied and these yield estimates that differ considerably. Quantification of the in vivo lytic capability of CTLs is essential for a detailed understanding of the immune response. This includes understanding the balance between viral replication and viral clearance, understanding the rate limiting steps in CTL killing and thus how killing can be increased and understanding the failure of CTL vaccines.

To date, the in vivo rate of CTL killing of virus-infected cells has been estimated in Lymphocytic Choriomeningitis Virus (LCMV) [1]–[5], Polyoma virus [6] and Human Immunodeficiency Virus Type 1/Simian Immunodeficiency Virus (HIV-1/SIV) [7]–[14]. These studies consistently find that CTL killing is considerably more rapid in LCMV and Polyoma virus than in HIV-1 infection (Table S1). In the LCMV system there are 5 studies of two data sets [1]–[5]. Both data sets were generated using a similar experimental approach in which labelled peptide-pulsed target cells were transferred into mice acutely or chronically infected with LCMV. It was found that target cells were killed by a single NP396 or GP276-specific CTL response, defined as a clone or clones specific for a single epitope, at a rate of 21–500 d^−1^ in acute infection and by either NP396- or GP33-specific CTL responses at a rate of 3–42.2 d^−1^ in chronic infection (Text S1). Even if we assume that there are no other effective CTL responses these estimates are extraordinarily high; in reality there are probably at least 2 or 3 (studies suggest 10 to 28 [15], [16]) other responses, yielding even higher estimates of killing attributable to the total CTL response, defined as all clones specific for a given virus. In Polyoma virus, using a similar experimental approach, killing rates of the same order of magnitude were found: a rate of 67.7 d^−1^ and 21.6 d^−1^ for a single MT389-specific response in acute and chronic infection respectively [6]. In HIV-1/SIV there are nine studies using three basic approaches. In the first approach [7], [8] the rate of CTL killing was estimated from the decline in cells productively infected with HIV-1 following reinfusion of ex vivo activated autologous HIV-1-specific CD8^+^ T cells. It was found that the killing rate attributable to the total CTL response was 4.4–9.8 d^−1^. This is almost certainly an overestimate of the rate of death attributable to natural CTL as it implies a death rate due to CTL that is higher than the total death rate of cells productively infected with HIV-1 [17]. In the second approach, the rate of CTL killing was estimated from the dynamics of HIV-1 escape from the CTL response in humans chronically infected with HIV-1 [9]. Here, much lower killing rates were calculated; it was estimated that a single CTL response is only responsible for about 2% of productively infected cell death, equivalent to a killing rate of 0.02 d^−1^ for a single response or 0.1–0.2 d^−1^ for the total response. This approach was later extended to primary HIV-1 infection [10] and SIV-1-infected macaques [11]–[14], yielding median estimates of 0.14 d^−1^ and 0.12 d^−1^ for a single CTL response respectively [10], [11]. Finally, indirect quantification of the contribution of CTL killing to total cell death in SIV-infection resulted in a killing rate of 0.3–0.4 d^−1^ for the total CTL response [18], [19].

Thus at one extreme, a target cell exposed to CTL specific for one LCMV peptide will have a lifespan (1/killing rate) of approximately 16 seconds; at the other extreme an HIV-1-infected cell that was killed by a CTL response against a single peptide would have a lifespan of nearly 2 months. Is it plausible that CD8^+^ T cell killing varies by 5 orders of magnitude between LCMV and HIV-1? If these are genuine differences, can we learn from the efficiency of the LCMV-specific response to therapeutically enhance the HIV-1-specific response? More generally, do these estimates span the physiological range, that is are they representative of the in vivo lytic action of CTL and thus define the maximal rate at which a virus can replicate and still be controlled or is the true physiological range even wider? With estimates for only three viruses it is impossible to answer these questions. Arguably, the estimates in LCMV may be higher than in the physiological system due to excess levels of peptide on the peptide-pulsed targets but conversely, CTL killing in HIV-1 infection may be abnormally low due to virus-induced damage to the immune system.

The aim of this paper is to estimate the rate of in vivo killing of CD8^+^ T cells in two distinct persistent virus infections: sheep infected with Bovine Leukemia Virus (BLV) and humans infected with Human T Lymphotropic Virus type 1 (HTLV-1).

## Results

### BLV model system

**Figure 1:**
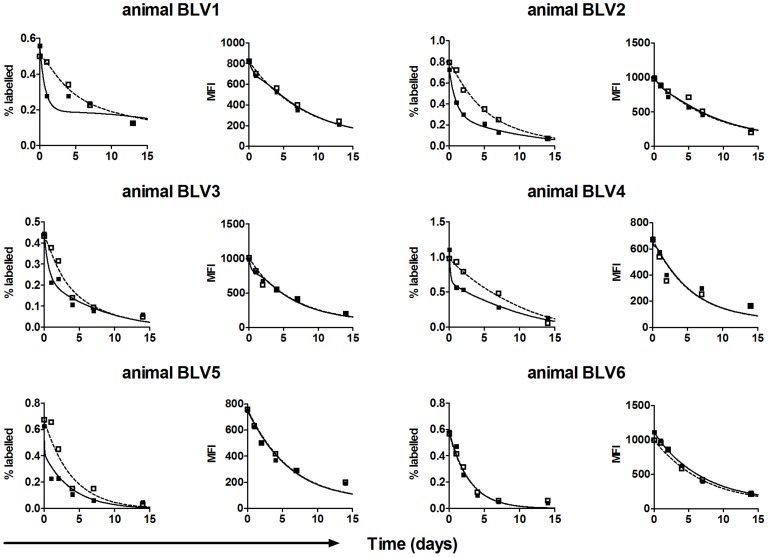
Experimental data of BLV infection and model fits. Percentage of B cells that were CFSE- and PKH26-positive (filled and open squares respectively), the MFI of CFSE and PKH26 fluorescence in label-positive B cells (filled and open squares respectively) and the model fits (solid and dashed lines respectively) for the six BLV infected animals. The data (but not the fits or analysis) have been published elsewhere [21].

**Figure 2:**
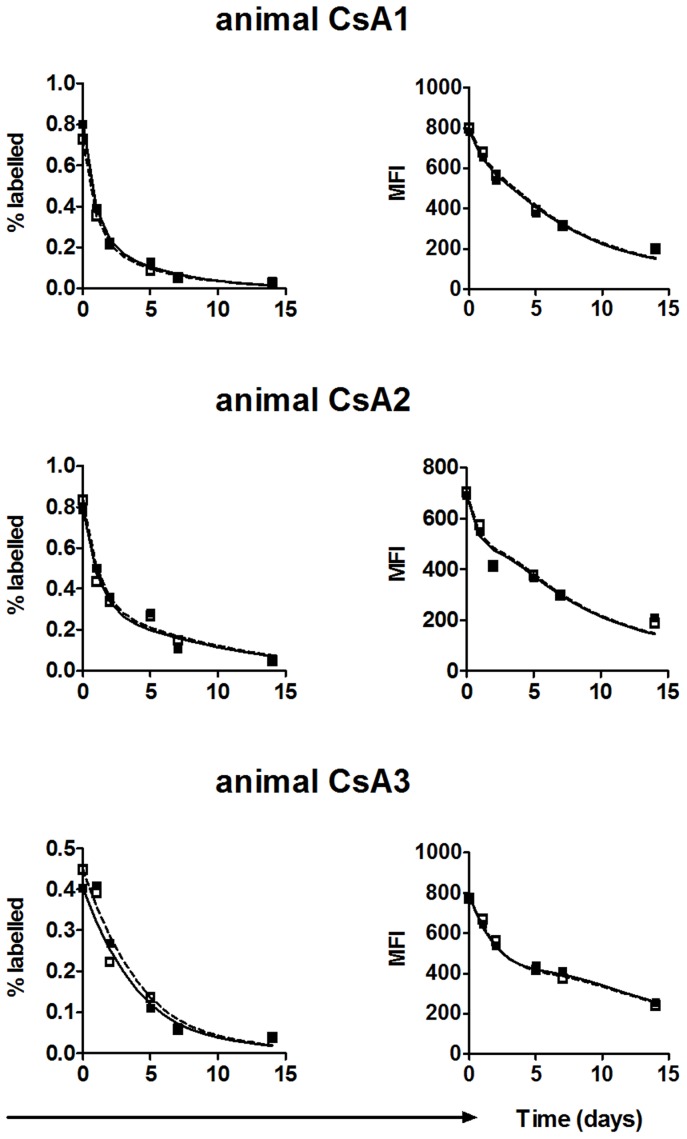
Experimental data of BLV infection and model fits. Percentage and MFI of the CFSE- and PKH26-positive B cell population (filled and open squares respectively) and the model fits (solid and dashed lines respectively) for the three CsA treated sheep. In this experiment the same animals were used as in the experiment without treatment, so CsA1 to CsA3 corresponds to BLV1 to BLV3. The data (but not the fits or analysis) have been published elsewhere [21]. A detail of the model fits of the first time point in given in Figure S2.

Bovine leukemia virus (BLV) is an exogenous retrovirus that naturally infects cows but which also establishes a persistent infection in B cells in sheep upon experimental infection [20]. Directly ex vivo most BLV-infected cells do not express viral protein. A more complete description of the experimental methods and resulting data is presented elsewhere [21]. Briefly, 6 BLV-infected and 3 uninfected sheep were studied. For each sheep, blood was collected and half the volume was incubated at 37°C for two hours while the other half was incubated at 4°C. The fractions were then labeled with carboxyfluorescein succinimidyl ester (CFSE) and PKH26 respectively, pooled and re-injected into the jugular vein. In the fraction that had been incubated at 37°C (CFSE-labelled) BLV-infected B cells expressed viral proteins, which was quantified by the up-regulation of the early viral protein Tax [21]. In contrast, cells incubated at 4°C (PKH26-labelled) did not express Tax. Blood was taken at multiple time points over a two week period and the fraction of labelled cells and the mean fluorescence intensity (MFI) of the label were measured. The proportion of CFSE-labelled cells consistently declined more rapidly than PKH26-labelled cells in the same sheep, except in animal BLV6 where no difference between the two cell populations was found (Figure 1). Swapping the labels (i.e. labelling cells incubated at 37°C with PKH26 and cells incubated at 4°C with CFSE) confirmed that it was the incubation temperature not the choice of label that determined the dynamics [21]. Furthermore, in uninfected sheep the loss of CFSE-labelled and PKH26-labelled cells was identical (Figure S1). The experiment was repeated in three animals injected intravenously with Cyclosporine A (CsA), a drug that interferes with TCR-mediated activation of the transcription factor NF-AT and subsequently with T cell activation, proliferation and the formation of the immunological synapse 22–26. Following CsA treatment, the CFSE-labelled and PKH26-labelled cells declined at similar rates (Figure 2). As CsA abrogates T cell function this implies that the majority of the excess loss of CFSE-labelled cells compared to PKH26-labelled cells could be attributed to CTL-mediated killing. We conclude that in the absence of CsA, CFSE-labelled cells are lost more rapidly than PKH26 labelled cells because CFSE-labelled cells expressed more viral protein and thus were more susceptible to CTL killing. In animal BLV6 proviral load is low, hence only a small fraction of CFSE-labelled cells are susceptible to CTL-killing resulting in an undetectable difference in loss of the two cell populations.

### Estimating rate of CTL killing of BLV-infected cells

We constructed a mathematical model to describe B cell dynamics in vivo. Three cell populations were considered: uninfected B cells, infected B cells that expressed viral proteins (henceforth called ag^+^) and silently-infected B cells that did not express viral proteins (henceforth ag^−^). We initially assume there are no ag^−^ cells labelled with CFSE at time zero; we later relax this assumption. Infected B cells that express viral proteins can be recognized and killed by CTL. Silent BLV-infected cells can up-regulate viral protein expression and thus become susceptible to CTL killing. The intensity of CFSE and PKH26 label is also modelled. The model parameters are the rate of CTL killing, the rate of up-regulation of viral proteins, the fraction of ag^+^ cells in the PKH26-labelled population at the start of the experiment and the proliferation rate and disappearance rate of the cell populations. There is evidence that Tax promotes cell proliferation [27], so the proliferation rates of the ag^+^ and ag^−^ populations were allowed to differ.

**Figure 3:**
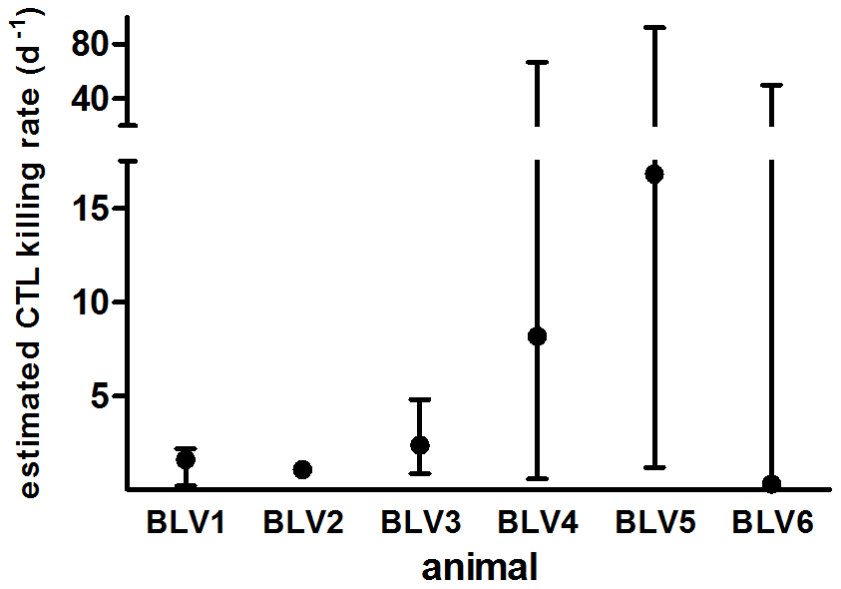
Estimates of CTL killing of ag^+^ B cells in 6 BLV-infected sheep, k (d^−1^) and their 95% confidence intervals. Three animals (BLV4–6) had large confidence intervals, partly due to lower proviral load in these animals. The median rate of killing is 1.98 d^−1^; if we limit our analysis to killing rates we can estimate with reasonable confidence then the median rate of killing is 1.60 d^−1^.

The fraction of infected cells in each animal was calculated from the proviral load (Methods and Table S2). The model was fitted to the data – the proportion of CFSE- and PKH26-labelled B cells and the MFI of CFSE and PKH26 label – using least squares regression. We estimated a median rate of killing by the total BLV-specific CTL response of ag^+^ BLV-infected cells of 1.98 d^−1^ (Table 1 and Figure 3). However, for three of the animals confidence intervals are large; this is partly because lower proviral load in these three animals makes the data less sensitive to changes in the killing rate. If we limit our analysis to killing rates we can estimate with reasonable confidence, i.e. estimates of animals BLV1–3, we find a median value of 1.60 d^−1^. Estimates of the other model parameters are provided in Table S3. The rate of killing in CsA-treated animals was non-zero (Table 1), though not all rates were significantly different from zero.

**Table 1:** Estimated killing rates in BLV infection.

Animal ID	k (d^−1^)
BLV1	1.60 (0.21–2.18)
BLV2	1.07 (0.69–1.52)
BLV3	2.36 (0.87–4.81)
BLV4	8.18 (0.56–66.58)
BLV5	16.82 (1.19–91.97)
BLV6	0.31 (0–49.98)
MEDIAN	1.98
CsA1	0 (0–1.39)
CsA2	1.24 (0.65–1.67)
CsA3	0.48 (0–1.55)
MEDIAN	1.14

Estimated killing rates of ag^+^ BLV-infected cells by the total BLV-specific CTL response; 95% confidence interval between brackets.

To test our model assumptions and the robustness of the estimates of CTL killing we considered a number of alternative models. Firstly, we relaxed the assumption that all CFSE-labelled infected B cells express viral proteins and instead we estimated the fraction of ag^+^ CFSE^+^ infected cells as a free parameter. We found that the fraction of ag^+^ CFSE^+^ infected cells was estimated to be ∼100% for all animals except BLV6, which has a very low proviral load, confirming our assumption (Table S5). Secondly, we tested a model in which we set the fraction of infected cells at the start of the experiment equal to the proviral load (rather than calculating it from the proviral load), but found that this gives a worse fit for most data sets (Table S6). Finally, we tested three further models representing, one with a damaged population that has a reduced lifespan due to the treatment, one with the same proliferation rate for ag^+^ and ag^−^ infected cells and one in which uninfected cells can become infected, which all yielded similar estimates of the rate of CTL killing (Text S2).

Killing is commonly considered to take place in organs like spleen and lymph nodes. Our model is based on the assumption that the balance between blood and lymphoid populations is established fast after reinjection of the labelled cells [28] compared to the timescale of the experimental sampling schedule so that the dynamics in the blood are representative of the dynamics in the organs. Fitting a model that includes the lymphoid compartment and the circulation to and from the blood is problematic; circulation parameters are highly dependent and this compartment was not measured experimentally. Nevertheless, we extended our model to include a lymphoid compartment. The rate at which cells leave the blood was fixed; multiple runs were performed with this fixed parameter taking different values from a realistic range. We found that the ratio of the concentration of lymphocytes in the blood to the concentration of lymphocytes in the lymphoid compartment and the time to steady state between these two compartments was of the order reported in the literature [28], [29] resulting in a median residence time in the lymphoid compartment of 2.6 h, which is at the faster end of the range reported in the literature. Forcing the residence time to 10 h does not have a large effect on either killing rate estimate or fit. Resulting killing estimates were very similar to what we found previously when we did not include the lymphoid compartment (Text S3 and Table S7).

### HTLV-1 system

Human T cell lymphotropic virus type 1 (HTLV-1) is a CD4^+^ T cell-tropic virus that establishes a persistent infection in humans. HTLV-1 is the etiological agent of a range of inflammatory diseases, of which HTLV-1-associated myelopathy/tropical spastic paraparesis (HAM/TSP), a chronic inflammation of the central nervous system, is best described. HAM/TSP is associated with a high HTLV-1 proviral load and so therapeutic intervention aims to reduce proviral load. One approach is to use the histone deacetylase inhibitor valproic acid (VPA) to activate HTLV-1 gene expression and expose infected cells to the host immune response thus reducing proviral load [30]. A more complete description of this approach and resulting data is presented elsewhere [30]. Briefly, 16 HAM/TSP patients were given VPA orally (20 mg/kg per day) and HTLV-1 proviral load was quantified at the start and after 1 and 3 months of treatment. We previously showed that, at physiological doses in vivo, VPA had no detectable impact on the efficiency of the CTL response though at higher doses in vitro a toxic effect has been reported [30]–[32].

### Estimating rate of CTL killing of HTLV-1-infected cells

**Figure 4:**
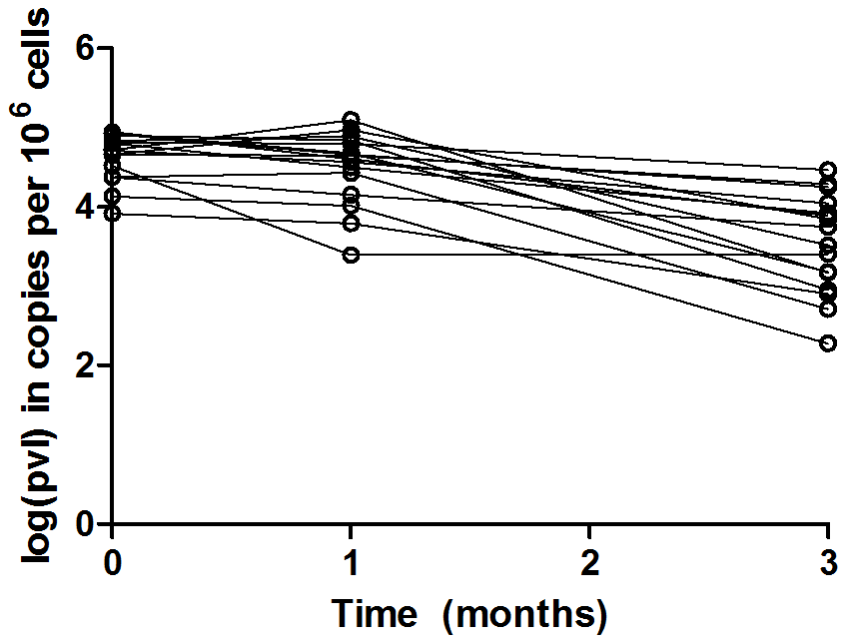
Proviral load and slope of decline of 16 HAM/TSP patients at the start of the VPA treatment and after three months. The data (but not the fits or analysis) have been published elsewhere [30].

Before VPA treatment, proviral load is relatively constant and most HTLV-1-infected cells do not express viral proteins [33], [34]. After VPA treatment, a consistent decline in proviral load was seen in all 16 HAM/TSP patients (Figure 4). VPA treatment does not affect the proliferation and intrinsic death rates of HTLV-1-infected CD4^+^ T cells [35], and NK cells are thought to play little cytotoxic role in the control of HTLV-1 infection [36] so we assume this decline is mainly due to the increased exposure of HTLV-1-infected CD4^+^ T cells to the CTL response. To estimate the CTL killing rate we converted proviral load to the fraction of infected cells (Methods and Table S2) and constructed a mathematical model describing the dynamics of infected cells after VPA-treatment. In the model the population of infected cells was divided into an ag^+^ and ag^−^ population. The ag^+^ population can be recognized and killed by CTL; the ag^−^ population can up-regulate viral protein expression and thus become susceptible to CTL killing. Both populations proliferate and have a CTL-independent death rate. We used estimates of proliferation and natural death rates from the literature. There are no quantitative estimates of how VPA increases the expression of viral proteins by HTLV-1 infected cells in vivo, so we assumed different values for the up-regulation rate (u) and estimated CTL killing rate (k) by fitting the model to the number of infected cells. We found that the value of k changes only minimally with the value of u. Only at very small values of u, when the availability of ag^+^ cells becomes limited by the slow up-regulation rate, do estimates of k change considerably, but in this range of u-values the quality of the fit, measured by the sum of squared residuals, is substantially worse (Figure S3). We found a median killing rate of 0.10 d^−1^ and a range of 0.03–0.13 d^−1^ (Table 2). This killing estimate is very similar if we assume the number of infected cells is equal to the proviral load (Table S8).

**Table 2:** Estimated range of killing rates in HTLV-1 infection.

	Estimated CTL killing rate (d^−1^)	
	Minimum	Maximum
DPK1	0.051	0.111
DPK2	0.034	0.094
DPK3	0.041	0.101
DPK4	0.047	0.107
DPK5	0.034	0.094
DPK6	0.042	0.102
DPK7	0.042	0.102
DPK8	0.058	0.118
DPK9	0.052	0.112
DPK10	0.067	0.127
DPK11	0.046	0.106
DPK12	0.074	0.134
DPK13	0.073	0.133
DPK14	0.069	0.129
DPK15	0.064	0.124
DPK16	0.053	0.113
MEDIAN	0.051	0.111

Estimated range of killing rates of ag^+^ HTLV-1-infected cells by the total HTLV-1-specific CTL response. The minimum and maximum estimates of CTL killing rate are calculated based on the minimum and maximum value of p_T_-d respectively.

## Discussion

The two systems we used to estimate the rate of CTL killing of virus-infected cells in vivo offer a number of benefits. Firstly, CTLs are unmanipulated and present in physiological quantities in the correct in vivo context. Secondly, targets are naturally infected cells. Finally, the large blood volume in sheep and humans permits repeat sampling as opposed to murine models, where it is usually necessary to perform serial sacrifices which introduces noise into the data. The limitations of this approach are the requirement to infer the dynamics of ag^+^ and ag^−^ cells from the total population of infected cells and the manipulation of the infected cells to induce viral protein expression.

In BLV-infected sheep we estimated the median killing rate of virus-expressing B cells by the total CTL response was 1.6 d^−1^. In HTLV-1-infected humans we estimated the median killing rate of virus-expressing CD4^+^ T cells by the total CTL response was 0.1 d^−1^.

**Figure 5:**
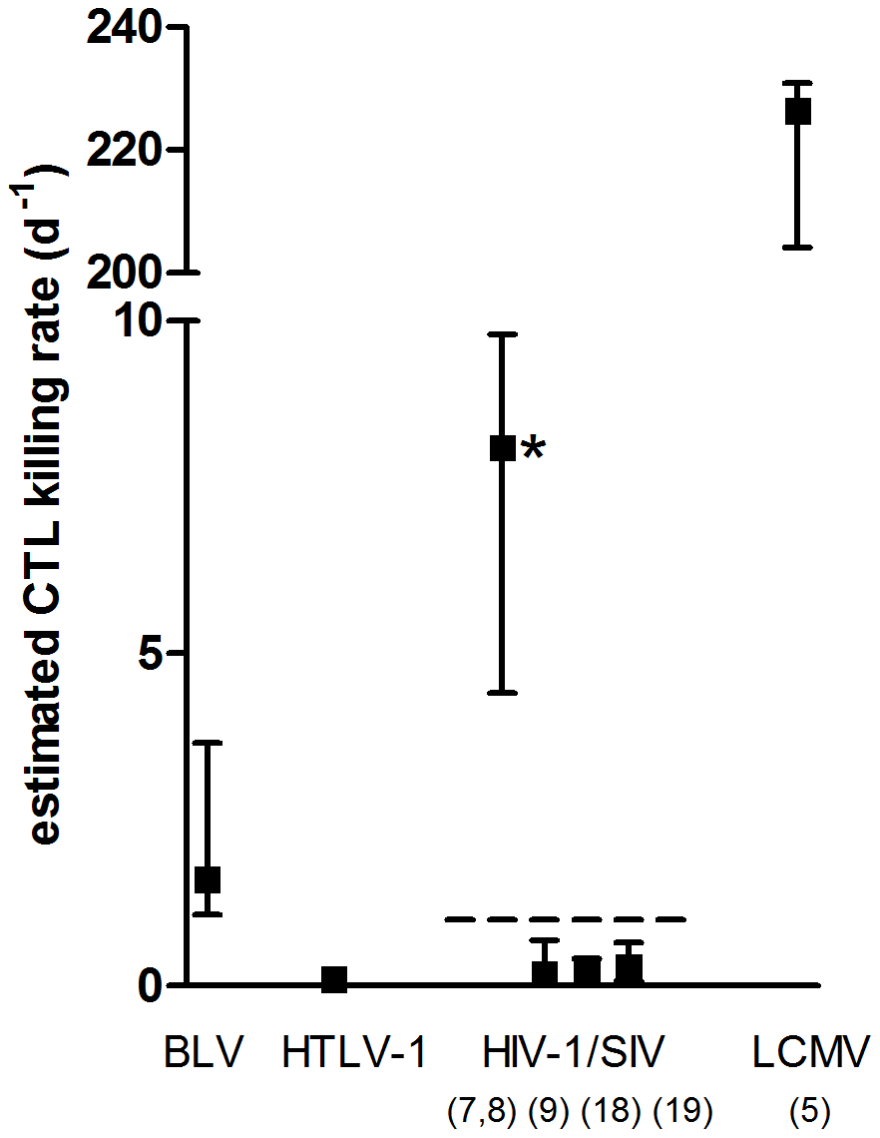
Range of killing estimates (d^−1^) of the total CTL response in persistent HTLV-1 and BLV infection compared to estimates in persistent HIV-1 and LCMV infection [2]–[5], [7], [9], [18], [19]. Median, minimum and maximum estimates are given. Numbers on x-axis refer to numbers in reference list. The killing estimate in LCMV-infection is based on a conservative estimate of 10 LCMV specific responses (Text S1). The HIV-1 estimate marked with * is almost certainly an overestimate as it implies a death rate due to CTL that is higher than the total death rate of cells productively infected with HIV-1 [17], indicated with the dashed line. Estimates of CTL killing in Polyoma virus infection and in SIV infection calculated from the dynamics of SIV escape [11]–[13] from one SIV-response are not included in this figure due to uncertainty about the total number of SIV-specific responses in the CTL response.

Previous estimates of the lytic potential of CD8^+^ T cells in persistent infection span a wide range, from 0.2 d^−1^ to 380 d^−1^ [5], [9]. Although both BLV and HTLV-1 infection are characterised by large expansions of virus-specific chronically activated CTL with immediate effector function ex vivo and no evidence of overt immune suppression [37], [38], our estimates are at the lower end of this range (Figure 5). This raises the possibility that the rate of CTL killing of targets in LCMV-infected mice is atypically high. In the LCMV studies the targets were peptide-pulsed splenocytes which are unlikely to represent naturally infected cells either in the levels of antigen presented or in frequency. However, Ganusov et al [6] found only 1–2 orders of magnitude between in vivo estimates of the killing rate of more physiological peptide concentrations and the maximal killing rate of peptide-pulsed target cells in Polyoma virus. Like many viruses, HIV-1, LCMV, HTLV-1 and BLV all employ strategies to evade the immune response e.g. [39], [40]; these strategies would not be utilised by peptide-pulsed targets, potentially leading to an overestimate of the true physiological rate of killing. It is likely that the estimate of CTL killing in HIV-1 infection, calculated by Wick et al [7], is also an overestimate of the natural killing rate of CD8^+^ T cells in the unmanipulated system because it is an order of magnitude higher than the total death rate attributable to all causes (including CD8^+^ T cell killing, activation induced cell death and any cytopathic effect of HIV-1), which has been estimated at 0.7–1 d^−1^ [17]. Given that the CTLs studied by Wick were expanded and activated in vitro it is not unlikely that they were more effective than in the absence of intervention. The large difference between these two killing rates and the rates found with more physiological target and effector cells raises the question if the total CTL response can potentially kill at high rates but in the physiological setting only realise a fraction of their maximum killing capacity, giving scope for the development of CTL based immunotherapy.

We estimated the CTL killing attributable to the total or single CTL response. Estimating the per capita CTL killing is non-trivial. Firstly it requires an estimate of the frequency of lytic CTL in lymphoid tissue (not necessarily the same as the frequency of IFNg secreting CD8+ T cells in the blood). More importantly it requires an assumption of the relationship between the rate of killing and the frequency of effectors and targets [4]. Therefore we do not attempt to express our estimates as a per-capita rate.

CTL killing in vitro has been quantified for HIV-1, LCMV and HTLV-1 [41]–[43]; the in vitro estimates differ considerably from the in vivo estimates but there is no systematic pattern in the discrepancy.

Unexpectedly, although the time course of CFSE and PHK26-labelled cells in CsA treated animals was virtually indistinguishable (Figure 2), the model fitting estimated a non-zero rate of killing of ag^+^ cells. This is because the loss of labelled cells is biphasic, i.e. we see an initially steep decline in labelled cells followed by a slower decline. In the simplified model we used, the only way in which cell loss can be biphasic is if ag^+^ and ag^−^ cells die at different rates and thus the model “forces” a non-zero estimate of CTL killing. The model could be extended to incorporate an alternative cause of biphasic decline. For instance if we assume a fraction of cells are damaged by the ex vivo treatment we find zero killing rates for two out of three CsA-treated animals and a lower killing rate for the third. For the untreated animals, this model gives similar estimates to the simplified model (Text S2). However, without a clear biological argument to extend the model in one way or another we felt it was preferable to use the simplified model.

Induction of viral protein expression will not only result in presentation of antigenic peptides on MHC; viral proteins will also be expressed on the surface of infected cells rendering them susceptible to antibody-dependent cytotoxicity. This mechanism of infected cell loss is not included in our model and our estimates of CTL killing rate may therefore represent an upper bound. However, in vitro studies have shown, for HTLV-1 that NK cells do not affect proviral load [36].

The estimates of proliferation rate we used to estimate killing rate in HTLV-1 infection were obtained using deuterated glucose labelling. Comparison of proliferation rates of T cell subsets estimated with either deuterated glucose or deuterated water has shown that the literature estimates of proliferation rate, measured with deuterated glucose, might be an overestimate [44], [45]. Consequently, HTLV-1 killing rates could be even lower than estimates presented in this study.

Recently, several studies found support for a non-lytic role for CD8^+^ T cells in viral infections [19], [46]–[49]. We assume that the anti-viral control we have quantified is largely lytic as both BLV and HTLV-1 spread mainly by mitotic replication and are therefore unlikely to be susceptible to non-lytic mechanisms.

In conclusion, the estimates we found for the CTL killing rate in BLV and HTLV-1 infection are at the lower end of the range described in the literature. This enables us to put current estimates into perspective and suggests that a CTL killing rate of the order of 1 per day or less, as found in HIV-1 and SIV, is not atypically low. The higher estimates are based on the loss of peptide-pulsed targets in LCMV and expanded, activated CD8^+^ T cells in HIV-1, both of which may overestimate the physiological rate of CTL killing. These estimates may represent the potential rather than the realised CTL efficiency. If this interpretation is correct there is considerable scope for improvement of the CTL response. Further studies in additional systems are urgently needed to test this suggestion.

## Methods

### Ethics statement

The animal protocol and experimental procedures in the BLV-study were approved by the Commission d'Ethique Animale affiliated to the Université de Liège (permit number 996) and were conducted in accordance with institutional and the European guidelines for animal care and use. The HTLV-1 study was approved by the Local and Regional Research Ethics Committee. All participants provided written informed consent and all procedures were carried out in accordance with the Declaration of Helsinki.

### Experiment design BLV-study

Blood was collected from six BLV-infected and three control sheep. Half of the volume was incubated at 37°C for 2 hours and labelled with CFSE, the other half was incubated at 4°C and labelled with PKH26. The two fractions were then pooled and intravenously re-injected. Blood was collected at different time points post-injection. The experiment was repeated with 3 BLV-infected sheep injected intravenously for 3 weeks with cyclosporin A at 5 mg/kg/day.

The percentage of CFSE and PKH26-positive B lymphocytes and the mean fluorescence intensity (MFI) of the positive and negative population were determined by flow cytometry. For each animal total number of B lymphocytes per µl of blood was determined. Proviral load (pvl) was measured in copies per number of B lymphocytes by real-time PCR.

Further details are provided in [21].

### Experiment design HTLV-1 study

Proviral load of HTLV-1 infected HAM/TSP patients was determined before and after one and three months of VPA treatment by real-time TaqMan PCR-method [50]. VPA was administered orally (20 mg/kg per day).

Further details are provided in [30].

### Fraction of infected cells

Proviral load quantifies the number of proviral copies rather than the number of infected cells. Due to the possibility of multiple infection these numbers are not necessarily equal. To estimate the fraction of infected cells from the proviral load we used an in silico approach. We assumed equal infection probability for each cell, whether already infected or uninfected, and set the total number of infection events (N) equal to pvl of each animal. For each animal we estimated the infected fraction by randomly “infecting” N cells in the total pool, allowing multiple infection, and counted the number of unique cells that were infected. We repeated this 50,000 times to acquire the mean and standard error of the infected fraction (Table S2).

### Modelling B lymphocyte population dynamics

Ganusov and de Boer [4] have convincingly argued that, in the absence of a detailed knowledge of the form of the CTL killing term it is preferable to estimate the overall rate of killing attributable to the CTL response rather than the per capita CTL killing rate as the latter is not robust to model assumptions. In LCMV-infection there are indications that the killing follows the law of mass action but it is unclear whether this applies to other infections and other hosts [51]; we therefore estimated the overall rate of killing. The experiment is conducted over 2 weeks so we do not model recirculation of cells from blood via the lymphoid organs as we assume the system reaches equilibrium on the timescale studied. Short-term incubation at 37°C of blood from BLV-infected sheep triggers viral expression [21]. We therefore assume that the CFSE-labelled B lymphocyte population can be divided into two subsets, one containing infected cells that express the viral protein (ag^+^, T) and one containing cells that are not infected (H). The PKH26-labelled population will consist of three different populations, one population of infected cells that do express the viral protein (T), one population of infected cells that do not express the virus (ag^−^, S) and one population of cells that are not infected (H); cells can move from the ag^−^ to the ag^+^ population at rate u.

The initial ratio between the ag^+^ (T) and ag^−^ (S) BLV –infected B lymphocytes in the PKH26-labelled population is a free parameter in the model.

We thus defined a model system describing the dynamics of each population:
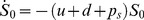
(1)

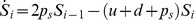
(2)

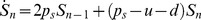
(3)

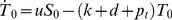
(4)

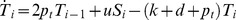
(5)

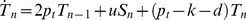
(6)

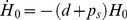
(7)

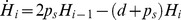
(8)

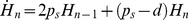
(9)Where n, is the number of divisions that an average CFSE- or PKH26-positive cell would have to undergo to become label-negative, i indicates the number of divisions a cell has already undergone, ranging from 1 to n-1, u is the rate at which infected ag^−^ cells, up-regulate viral protein, p_s_ is the proliferation rate of ag^−^ cells and uninfected cells, p_t_ is the proliferation rate of ag^+^ cells, d is the disappearance rate, and k is the rate at which ag^+^ cells are killed by CTL (all rates in d^−1^).

To determine n we used the method in reference [52]:
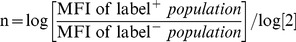
(10)and found that on average after 6 divisions the label becomes undetectable (Table S9).

The predicted fraction of labelled cells is calculated by summing the cells in S_i_, T_i_ and H_i_ and dividing this by the total number of B lymphocytes for each sheep. The predicted MFI was:
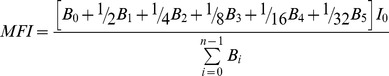
(11)Where B is T+S+H and I_0_ is the MFI of the label-positive population at the start of the experiment.

In a previous publication [21], we used a simple descriptive model to analyze the disappearance rate (as opposed to CTL killing rate) of the total population of B cells (as opposed to the ag^+^ population of BLV infected B cells). Here we use mechanistic models to estimate the rate of CD8^+^ T cell killing.

### Model fitting

The data (fraction of labelled cells and the MFI) was scaled to obtain equal means. The model was fitted using the function modFit in the package FME in R [53]. The confidence interval on the estimates was obtained by bootstrapping the cases and trimming the extremes [54], [55].

### Estimating CD8^+^ T cell killing in HTLV-1 infection

To estimate CD8^+^ T cell killing in HTLV-1 infection from the decline in number of infected cells we used a model describing the change in number of ag^−^ infected cells (S) and ag^+^ infected cells (T), together representing the change in number of infected cells (N):
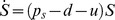
(12)

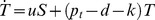
(13)

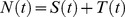
(14)where p_s_ and p_t_ are proliferation rates of the ag^−^ and ag^+^ population of infected cells respectively, d is the CTL-independent, intrinsic death rate, u is the upregulation rate of viral proteins and k is CTL killing rate of the total HTLV-1 specific CD8^+^ T cell population. In the literature p_t_ has been estimated as 0.07–0.13 d^−1^ [56] and p_s_ as 0.027 d^−1^ [45], [57]. We set d equal to p_s_ and estimated k from these values over a range of values for u. We repeated this procedure using proviral load instead of the fraction of infected cells and found similar k estimates (Table S8).

## Supporting Information

Figure S1 Experimental data and model fits of control animals. Percentage of B cells that were CFSE- and PKH26-positive (open and filled squares respectively), the MFI of CFSE and PKH26 fluorescence in label-positive B cells (open and filled squares respectively) and the model prediction (solid and dashed lines respectively) for the three non-infected control animals.
(TIF)

Figure S2 Experimental data of BLV infection and model fits of the first time point. Percentage of B cells that were CFSE- and PKH26-positive (filled and open squares respectively), the MFI of CFSE and PKH26 fluorescence in label-positive B cells (filled and open squares respectively) and the model fits (solid and dashed lines respectively) for the six BLV infected animals.
(TIF)

Figure S3 Effect of upregulation rate (u) of viral protein expression after vpa-treatment on killing estimate (k). Killing estimates (open symbols) and sum of squared residuals (closed symbols) resulting from model fits using different values of u for the 16 HAM/TSP HTLV-1 infected patients in the VPA-experiment. The value of k changes only minimally with the value of u. Only at very small values of u, estimates of k change considerably, but in this range of u-values the quality of the fit, measured by the sum of squared residuals, is substantially worse.
(TIF)

Table S1 Estimates of CTL killing in the literature in both the reported units and converted to consistent units.
(DOCX)

Table S2 Estimated fraction of cells that are infected (F) for A) B cells in BLV infection and B) T cells in HTLV-1 infection. The fraction was estimated based on proviral load (pvl in copies/cell) and the assumption of equal infection probability for all cells (methods). Mean estimated infected fraction (F) and standard deviation (SD) are given.
(DOCX)

Table S3 Estimates of killing rate (k), death rate (d), fraction of Tax-negative infected cells (f), proliferation rate (p) for ag^+^ and ag^−^ populations, and transition rate (u) per day for each BLV-infected animal (BLV1 to BLV6), the non-infected animals (NI1 to NI3) and the CsA-treated animals (CsA1 to CsA3).
(DOCX)

Table S4 Mean of the sensitivity function of the model parameters for each BLV-infected animal (BLV1 to BLV6).
(DOCX)

Table S5 Parameter estimates of the model in which the fraction of ag^+^ CFSE^+^ infected cells (f-CFSE^+^) is estimated as a free parameter. Negative ΔAIC_c_ indicates a better fit for the model with f-CFSE^+^ = 1, positive ΔAIC_c_ indicates a better fit when f-CFSE^+^ is fitted as a free parameter. Other parameters are killing rate (k), death rate (d), fraction of Tax-positive infected cells (f), proliferation rate (p) for ag^+^ and ag^−^ populations, and transition rate (u) per day for each BLV-infected animal (BLV1 to BLV6).
(DOCX)

Table S6 Sum of Squared Residuals (SSR) for the original model, in which the fraction of infected cells is inferred from proviral load (Table S2), and a model in which the fraction of infected cells is set equal to proviral load. Both models have the same number of parameters so AIC_c_ would directly reflect SSR. In general a lower SSR is found for the original model, especially when the difference between inferred fraction of infected cells and proviral load is large.
(DOCX)

Table S7 Mean killing rate per animal at different values of h fixed (min and max found at different values of h) in BLV infection estimated by the circulation model.
(DOCX)

Table S8 Comparison of the killing rate (k) estimated using the fraction of infected cells calculated from proviral load (methods) with that estimated if we assume the number of infected cells is equal to the proviral load. These estimates are based on literature estimates for proliferation rate of the ag^+^ population p_T_ = 0.10 and of the ag^−^ population p_S_ = 0.027 d^−1^ and the assumption that death rate d equals p_S_.
(DOCX)

Table S9 Average number of divisions that an average CFSE- or PKH26-positive cell would have to undergo to become label-negative.
(DOCX)

Text S1 Other estimates of CTL killing from the literature.
(DOCX)

Text S2 Additional models.
(DOCX)

Text S3 Circulation model.
(DOCX)
