# Supplementary figures and images for: Rates of CTL Killing in Persistent Viral Infection In Vivo
Source: PLoS Comput Biol. 2014 Apr 3;10(4):e1003534. doi: 10.1371/journal.pcbi.1003534 (PMC3974637; doi:10.1371/journal.pcbi.1003534)

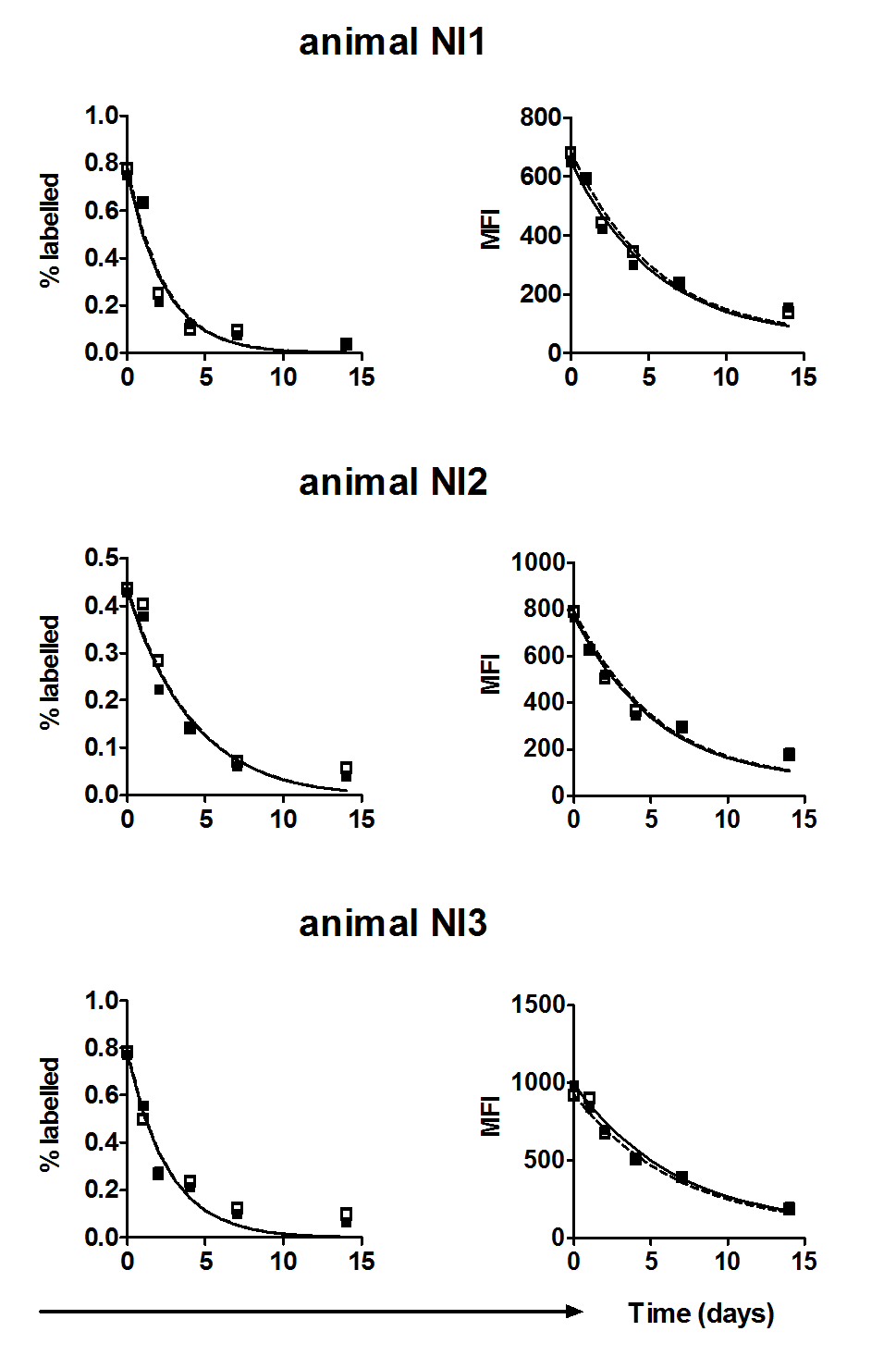

Supplement: Figure S1 — Experimental data and model fits of control animals. Percentage of B cells that were CFSE- and PKH26-positive (open and filled squares respectively), the MFI of CFSE and PKH26 fluorescence in label-positive B cells (open and filled squares respectively) and the model prediction (solid and dashed lines respectively) for the three non-infected control animals. (TIF) [file pcbi.1003534.s001.tif]

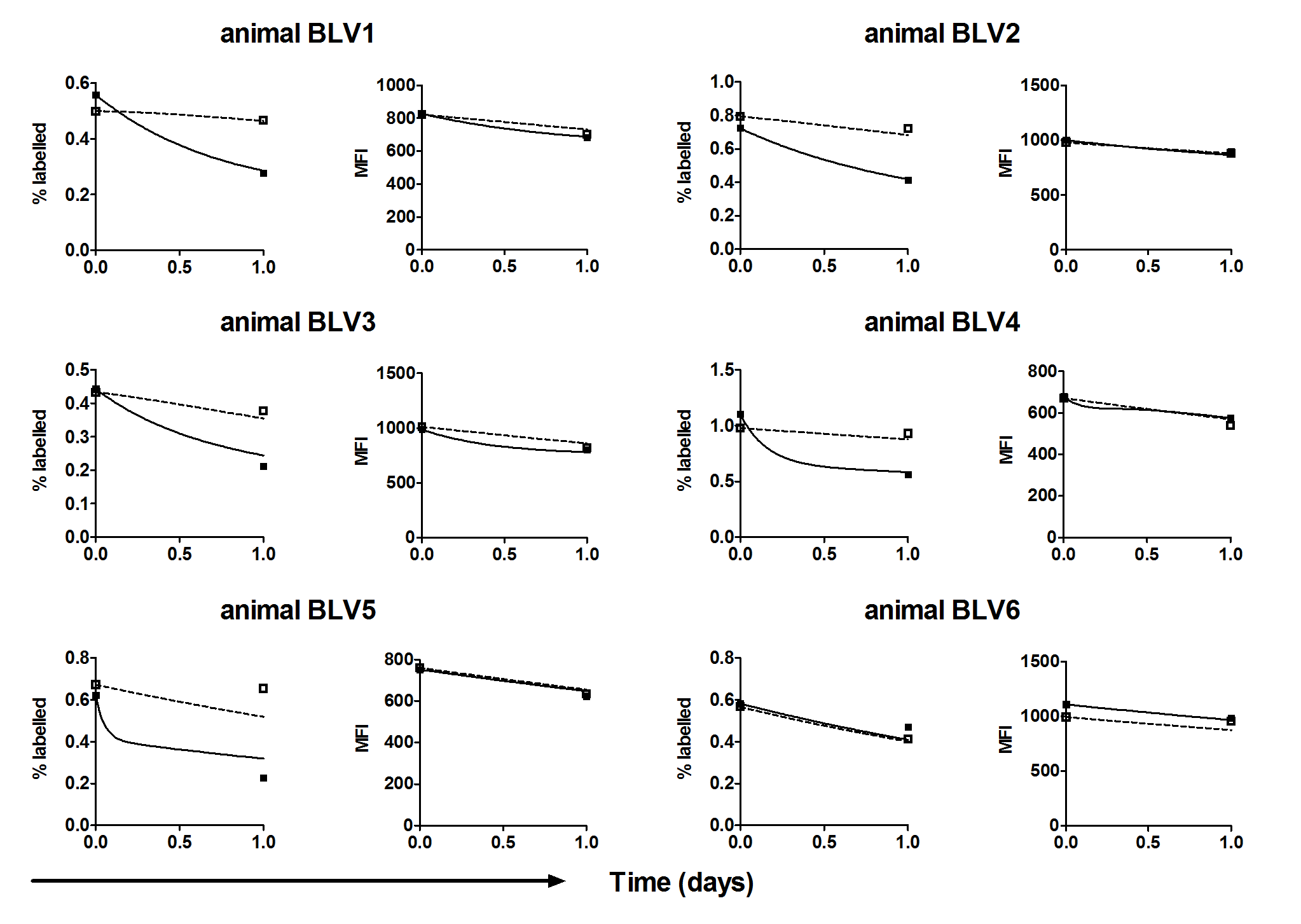

Supplement: Figure S2 — Experimental data of BLV infection and model fits of the first time point. Percentage of B cells that were CFSE- and PKH26-positive (filled and open squares respectively), the MFI of CFSE and PKH26 fluorescence in label-positive B cells (filled and open squares respectively) and the model fits (solid and dashed lines respectively) for the six BLV infected animals. (TIF) [file pcbi.1003534.s002.tif]

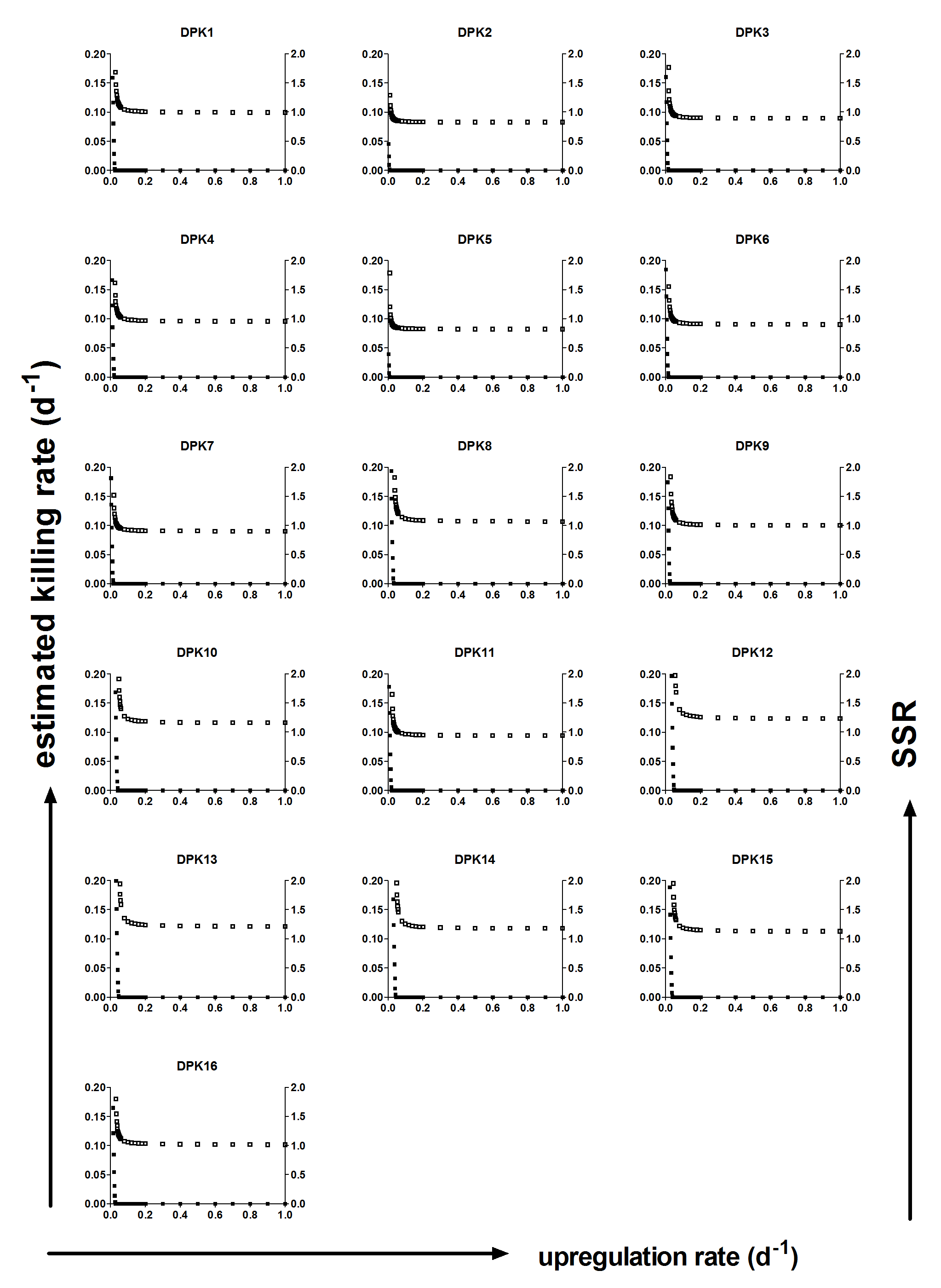

Supplement: Figure S3 — Effect of upregulation rate (u) of viral protein expression after vpa-treatment on killing estimate (k). Killing estimates (open symbols) and sum of squared residuals (closed symbols) resulting from model fits using different values of u for the 16 HAM/TSP HTLV-1 infected patients in the VPA-experiment. The value of k changes only minimally with the value of u. Only at very small values of u, estimates of k change considerably, but in this range of u-values the quality of the fit, measured by the sum of squared residuals, is substantially worse. (TIF) [file pcbi.1003534.s003.tif]
