# Supplementary material for: Rates of CTL Killing in Persistent Viral Infection In Vivo
Source: PLoS Comput Biol. 2014 Apr 3;10(4):e1003534. doi: 10.1371/journal.pcbi.1003534 (PMC3974637; doi:10.1371/journal.pcbi.1003534)
Supplement: Table S1 — Estimates of CTL killing in the literature in both the reported units and converted to consistent units. (DOCX) [file pcbi.1003534.s004.docx]

| Reporting paper | System | Killing rate as reported  (units vary) | Overall killing rate  (per day) | |
| --- | --- | --- | --- | --- |
|  |  |  | Single CTL  response | Total CTL  response |
| ([3](#_ENREF_3)) | Acute LCMV NP396 | 1.33 per minute per CTL | 126.4 |  |
|  | Acute LCMV GP276 | 0.70 per minute per CTL | 21.2 |  |
| ([4](#_ENREF_4)) | Acute LCMV NP396 | 3.71 per minute per CTL | 352.6 |  |
|  | Acute LCMV GP276 | 2.19 per minute per CTL | 66.2 |  |
| ([5](#_ENREF_5)) | Acute LCMV NP396 | Half-life 2 minutes | 499.1 |  |
|  | Acute LCMV GP276 | Half-life 14 minutes | 71.3 |  |
| ([6](#_ENREF_6)) | Chronic LCMV NP396  Median of 3 cases | Half-life 328.9 minutes | 3.0 | 200.5 |
|  | Chronic LCMV GP33  Median of 3 cases | Half-life 23.63 minutes | 42.2 |  |
| ([9](#_ENREF_9)) | Acute Polyoma virus | 4.16 per minute per CTL | 67.7 |  |
|  | Chronic Polyoma virus | 1.90 per minute per CTL | 21.6 |  |
| ([10](#_ENREF_10)) | Chronic HIV-1 Patient 1 | 0.14 μl cell^-1^d^-1^ |  | 8.1 |
|  | Chronic HIV Patient 2 | 0.17 μl cell^-1^d^-1^ |  | 9.8 |
|  | Chronic HIV Patient 3 | 0.076 μl cell^-1^d^-1^ |  | 4.4 |
| ([12](#_ENREF_12)) | Late primary/Chronic HIV-1.  Median of 21 cases. | Single response 0.02d^-1^, total response 0.2d^-1^ | 0.02 | 0.2 |
| ([13](#_ENREF_13)) | Acute HIV-1.  Median of 15 cases. | Single response 0.14d^-1^ | 0.14 | <0.8 |
| ([14](#_ENREF_14)) | Acute SIV infection.  Single estimate for 4 cases. | 0.016 μl cell^-1^d^-1^ | 0.7 |  |
| ([15](#_ENREF_15)) | Acute SHIV.  Median of 4 cases | Single response 0.41d^-1^ | 0.41 |  |
| ([16](#_ENREF_16)) | Acute and chronic SIV | Single response 0.12d^-1^ | 0.12 |  |
| ([17](#_ENREF_17)) | Acute/Chronic SIV  Mean of 11 cases. | Total CTL response responsible for 41.8% of total infected cell death. |  | 0.42 |
| ([19](#_ENREF_19)) | Acute/Chronic SIV  Mean of 9 cases. | Total response 0.3 d^-1^ |  | 0.3 |

**Table S1. Estimates of CTL killing in the literature in both the reported units and converted to consistent units.**
