# Supplementary material for: Rates of CTL Killing in Persistent Viral Infection In Vivo
Source: PLoS Comput Biol. 2014 Apr 3;10(4):e1003534. doi: 10.1371/journal.pcbi.1003534 (PMC3974637; doi:10.1371/journal.pcbi.1003534)
Supplement: Table S2 — Estimated fraction of cells that are infected (F) for A) B cells in BLV infection and B) T cells in HTLV-1 infection. The fraction was estimated based on proviral load (pvl in copies/cell) and the assumption of equal infection probability for all cells (methods). Mean estimated infected fraction (F) and standard deviation (SD) are given. (DOCX) [file pcbi.1003534.s005.docx]

| A. | | animal ID | | Pvl | | F | | | | SEM | |  |  |  |  |
| --- | --- | --- | --- | --- | --- | --- | --- | --- | --- | --- | --- | --- | --- | --- | --- |
|  | | BLV1 | | 1.061 | | 0.654 | | | | 0.004 | |  |  |  |  |
|  | | BLV2 | | 0.900 | | 0.593 | | | | 0.004 | |  |  |  |  |
|  | | BLV3 | | 0.633 | | 0.469 | | | | 0.004 | |  |  |  |  |
|  | | BLV4 | | 0.534 | | 0.414 | | | | 0.004 | |  |  |  |  |
|  | | BLV5 | | 0.413 | | 0.338 | | | | 0.003 | |  |  |  |  |
|  | | BLV6 | | 0.070 | | 0.067 | | | | 0.001 | |  |  |  |  |
| B. | |  | | before VPA treatment | | | | |  |  | | before VPA treatment | | | |
|  | | Patient ID | | pvl | | F | | SD |  | Patient ID | | pvl | | F | SD |
|  | | DPK1 | | 8.31E-03 | | 8.28E-03 | | 5.84E-06 |  | DPK9 | | 8.73E-02 | | 8.36E-02 | 5.74E-05 |
|  | | DPK2 | | 4.57E-02 | | 4.47E-02 | | 3.11E-05 |  | DPK10 | | 2.39E-02 | | 2.37E-02 | 1.66E-05 |
|  | | DPK3 | | 2.39E-02 | | 2.36E-02 | | 1.66E-05 |  | DPK11 | | 4.60E-02 | | 4.50E-02 | 3.13E-05 |
|  | | DPK4 | | 6.32E-02 | | 6.13E-02 | | 4.26E-05 |  | DPK12 | | 8.01E-02 | | 7.70E-02 | 5.27E-05 |
|  | | DPK5 | | 6.58E-02 | | 6.37E-02 | | 4.40E-05 |  | DPK13 | | 1.36E-02 | | 1.35E-02 | 9.41E-06 |
|  | | DPK6 | | 8.36E-02 | | 8.02E-02 | | 5.51E-05 |  | DPK14 | | 8.49E-02 | | 8.14E-02 | 5.61E-05 |
|  | | DPK7 | | 5.13E-02 | | 5.00E-02 | | 3.49E-05 |  | DPK15 | | 5.20E-02 | | 5.07E-02 | 3.51E-05 |
|  | | DPK8 | | 6.79E-02 | | 6.57E-02 | | 4.54E-05 |  | DPK16 | | 3.31E-02 | | 3.25E-02 | 2.29E-05 |
|  | |  | |  | |  | |  |  |  | |  | |  |  |
|  | |  | | after 1 month of VPA treatment | | | | |  |  | | after 1 month of VPA treatment | | | |
|  | | Patient ID | | Pvl | | F | | SD |  | Patient ID | | pvl | | F | SD |
|  | | DPK1 | | 6.20E-03 | | 6.18E-03 | | 4.35E-06 |  | DPK9 | | 4.02E-02 | | 3.94E-02 | 2.75E-05 |
|  | | DPK2 | | 4.46E-02 | | 4.36E-02 | | 3.03E-05 |  | DPK10 | | 2.69E-02 | | 2.65E-02 | 1.85E-05 |
|  | | DPK3 | | 1.42E-02 | | 1.41E-02 | | 1.00E-05 |  | DPK11 | | 9.31E-02 | | 8.89E-02 | 6.11E-05 |
|  | | DPK4 | | 3.13E-02 | | 3.08E-02 | | 2.15E-05 |  | DPK12 | | 6.98E-02 | | 6.74E-02 | 4.66E-05 |
|  | | DPK5 | | 6.25E-02 | | 6.06E-02 | | 4.19E-05 |  | DPK13 | | 1.04E-02 | | 1.03E-02 | 7.26E-06 |
|  | | DPK6 | | 4.59E-02 | | 4.48E-02 | | 3.12E-05 |  | DPK14 | | 4.77E-02 | | 4.66E-02 | 3.25E-05 |
|  | | DPK7 | | 3.69E-02 | | 3.62E-02 | | 2.53E-05 |  | DPK15 | | 1.25E-01 | | 1.18E-01 | 7.95E-05 |
|  | | DPK8 | | 7.81E-02 | | 7.51E-02 | | 5.17E-05 |  | DPK16 | | 2.47E-03 | | 2.46E-03 | 1.73E-06 |
|  | |  | |  | |  | |  |  |  | |  | |  |  |
|  | |  | | after 3 months of VPA treatment | | | | |  |  | | after 3 months of VPA treatment | | | |
|  | | Patient ID | | Pvl | | F | | SD |  | Patient ID | | pvl | | F | SD |
|  | | DPK1 | | 7.95E-04 | | 7.95E-04 | | 5.64E-07 |  | DPK9 | | 7.79E-03 | | 7.76E-03 | 5.45E-06 |
|  | | DPK2 | | 1.94E-02 | | 1.92E-02 | | 1.35E-05 |  | DPK10 | | 5.17E-04 | | 5.17E-04 | 3.65E-07 |
|  | | DPK3 | | 5.62E-03 | | 5.60E-03 | | 3.95E-06 |  | DPK11 | | 7.01E-03 | | 6.98E-03 | 4.92E-06 |
|  | | DPK4 | | 8.39E-03 | | 8.35E-03 | | 5.90E-06 |  | DPK12 | | 8.97E-04 | | 8.97E-04 | 6.34E-07 |
|  | | DPK5 | | 2.92E-02 | | 2.88E-02 | | 2.02E-05 |  | DPK13 | | 1.89E-04 | | 1.89E-04 | 1.32E-07 |
|  | | DPK6 | | 1.79E-02 | | 1.77E-02 | | 1.25E-05 |  | DPK14 | | 1.52E-03 | | 1.52E-03 | 1.08E-06 |
|  | | DPK7 | | 1.11E-02 | | 1.10E-02 | | 7.79E-06 |  | DPK15 | | 1.48E-03 | | 1.48E-03 | 1.05E-06 |
|  | | DPK8 | | 3.28E-03 | | 3.27E-03 | | 2.31E-06 |  | DPK16 | | 2.56E-03 | | 2.56E-03 | 1.80E-06 |
