# Supplementary material for: Rates of CTL Killing in Persistent Viral Infection In Vivo
Source: PLoS Comput Biol. 2014 Apr 3;10(4):e1003534. doi: 10.1371/journal.pcbi.1003534 (PMC3974637; doi:10.1371/journal.pcbi.1003534)
Supplement: Table S3 — Estimates of killing rate (k), death rate (d), fraction of Tax-negative infected cells (f), proliferation rate (p) for ag+ and ag− populations, and transition rate (u) per day for each BLV-infected animal (BLV1 to BLV6), the non-infected animals (NI1 to NI3) and the CsA-treated animals (CsA1 to CsA3). (DOCX) [file pcbi.1003534.s006.docx]

| **Animal ID** | k | d | f | p ag^+^ | p ag^─^ | u |
| --- | --- | --- | --- | --- | --- | --- |
| BLV1 | 1.60 | 0.12 | 1.1E-9 | 0.36 | 0.11 | 0.23 |
| BLV2 | 1.07 | 0.20 | 0.06 | 0.23 | 0.10 | 0.17 |
| BLV3 | 3.66 | 0.27 | 8.5E-9 | 8.56 | 0.14 | 0.37 |
| BLV4 | 8.01 | 0.24 | 0.04 | 16.32 | 0.15 | 0.10 |
| BLV5 | 37.94 | 0.41 | 0 | 0 | 0.15 | 1.9E-7 |
| BLV6 | 1.48 | 0.47 | 0 | 4.92 | 0.13 | 2.3E-9 |
| NI1 | - | 0.58 | - | - | 0.16 | - |
| NI2 | - | 0.41 | - | - | 0.17 | - |
| NI3 | - | 0.52 | - | - | 0.14 | - |
| CsA1 | 1.14 | 0.33 | 0.98 | 0.29 | 0.13 | 1 |
| CsA2 | 1.25 | 0.22 | 1 | 0.55 | 0.12 | 0 |
| CsA3 | 0.48 | 0.25 | 0.73 | 0.44 | 0.08 | 1 |
