# Supplementary material for: Rates of CTL Killing in Persistent Viral Infection In Vivo
Source: PLoS Comput Biol. 2014 Apr 3;10(4):e1003534. doi: 10.1371/journal.pcbi.1003534 (PMC3974637; doi:10.1371/journal.pcbi.1003534)
Supplement: Table S4 — Mean of the sensitivity function of the model parameters for each BLV-infected animal (BLV1 to BLV6). (DOCX) [file pcbi.1003534.s007.docx]

|  | d | f | k | p ag^+^ | p ag^—^ | u |
| --- | --- | --- | --- | --- | --- | --- |
| BLV1 | 0.339 | 0.000 | 0.033 | 0.003 | 0.000 | 0.062 |
| BLV2 | 0.577 | 0.004 | 0.037 | 0.002 | 0.000 | 0.049 |
| BLV3 | 0.796 | 0.000 | 0.012 | 0.008 | 0.000 | 0.033 |
| BLV4 | 4.598 | 0.000 | 4.004 | 0.854 | 0.010 | 0.079 |
| BLV5 | 5.488 | 0.000 | 40.754 | 30.101 | 0.153 | 0.000 |
| BLV6 | 1.354 | 0.000 | 0.003 | 0.005 | 0.000 | 0.000 |
