# Supplementary material for: Rates of CTL Killing in Persistent Viral Infection In Vivo
Source: PLoS Comput Biol. 2014 Apr 3;10(4):e1003534. doi: 10.1371/journal.pcbi.1003534 (PMC3974637; doi:10.1371/journal.pcbi.1003534)
Supplement: Table S5 — Parameter estimates of the model in which the fraction of ag+ CFSE+ infected cells (f-CFSE+) is estimated as a free parameter. Negative ΔAICc indicates a better fit for the model with f-CFSE+ = 1, positive ΔAICc indicates a better fit when f-CFSE+ is fitted as a free parameter. Other parameters are killing rate (k), death rate (d), fraction of Tax-positive infected cells (f), proliferation rate (p) for ag+ and ag− populations, and transition rate (u) per day for each BLV-infected animal (BLV1 to BLV6). (DOCX) [file pcbi.1003534.s008.docx]

| **animal ID** | **k** | **d** | **f-PKH^+^** | **f-CFSE^+^** | **p ag^+^** | **p ag^-^** | **u** | **ΔAICc** |
| --- | --- | --- | --- | --- | --- | --- | --- | --- |
| BLV1 | 5.470 | 0.138 | 1.1E-09 | **0.643** | 3 | 0.109 | 0.139 | 10.48 |
| BLV2 | 1.309 | 0.208 | 0.028 | **0.889** | 0.260 | 0.102 | 0.146 | -5.48 |
| BLV3 | 4.155 | 0.276 | 4.4E-10 | **0.948** | 3 | 0.144 | 0.300 | -4.71 |
| BLV4 | 7.994 | 0.269 | 0 | **1** | 3 | 0.164 | 0.013 | -5.60 |
| BLV5 | 10 | 0.408 | 0 | **1** | 0.037 | 0.149 | 0 | -5.29 |
| BLV6 | 0.474 | 0.466 | 1.1E-07 | **2.1E-08** | 0 | 0.129 | 0.619 | -5.84 |
