# Supplementary material for: Rates of CTL Killing in Persistent Viral Infection In Vivo
Source: PLoS Comput Biol. 2014 Apr 3;10(4):e1003534. doi: 10.1371/journal.pcbi.1003534 (PMC3974637; doi:10.1371/journal.pcbi.1003534)
Supplement: Table S6 — Sum of Squared Residuals (SSR) for the original model, in which the fraction of infected cells is inferred from proviral load (Table S2), and a model in which the fraction of infected cells is set equal to proviral load. Both models have the same number of parameters so AICc would directly reflect SSR. In general a lower SSR is found for the original model, especially when the difference between inferred fraction of infected cells and proviral load is large. (DOCX) [file pcbi.1003534.s009.docx]

|  | SSR | |
| --- | --- | --- |
|  | original model | fraction infected cells = pvl |
| BLV1 | 0.016 | 0.049 |
| BLV2 | 0.014 | 0.025 |
| BLV3 | 0.011 | 0.010 |
| BLV4 | 0.099 | 0.100 |
| BLV5 | 0.051 | 0.044 |
| BLV6 | 0.014 | 0.014 |
| CsA1 | 0.004 | 0.024 |
| CsA2 | 0.031 | 0.055 |
| CsA3 | 0.016 | 0.020 |
