# Supplementary material for: Rates of CTL Killing in Persistent Viral Infection In Vivo
Source: PLoS Comput Biol. 2014 Apr 3;10(4):e1003534. doi: 10.1371/journal.pcbi.1003534 (PMC3974637; doi:10.1371/journal.pcbi.1003534)
Supplement: Table S7 — Mean killing rate per animal at different values of h fixed (min and max found at different values of h) in BLV infection estimated by the circulation model. (DOCX) [file pcbi.1003534.s010.docx]

| **Animal ID** | **k (d^-1^)** |
| --- | --- |
| BLV1 | 0.74 (0.01-1.75) |
| BLV2 | 1.74 (0.82-1.81) |
| BLV3 | 1.73 (1.66-1.77) |
| BLV4 | 1.63 (0.54-1.66) |
| BLV5 | 1.65 (1.38-1.65) |
| BLV6 | 0.46 (0.39-1.04) |
| MEDIAN | 1.64 |
