# Supplementary material for: Rates of CTL Killing in Persistent Viral Infection In Vivo
Source: PLoS Comput Biol. 2014 Apr 3;10(4):e1003534. doi: 10.1371/journal.pcbi.1003534 (PMC3974637; doi:10.1371/journal.pcbi.1003534)
Supplement: Table S8 — Comparison of the killing rate (k) estimated using the fraction of infected cells calculated from proviral load (methods) with that estimated if we assume the number of infected cells is equal to the proviral load. These estimates are based on literature estimates for proliferation rate of the ag+ population pT = 0.10 and of the ag− population pS = 0.027 d−1 and the assumption that death rate d equals pS. (DOCX) [file pcbi.1003534.s011.docx]

|  | k estimate (d^-1^) | |
| --- | --- | --- |
|  | Number of infected cells | pvl |
| DPK1 | 0.099 | 0.100 |
| DPK2 | 0.083 | 0.083 |
| DPK3 | 0.089 | 0.089 |
| DPK4 | 0.096 | 0.096 |
| DPK5 | 0.082 | 0.082 |
| DPK6 | 0.090 | 0.090 |
| DPK7 | 0.090 | 0.090 |
| DPK8 | 0.107 | 0.107 |
| DPK9 | 0.100 | 0.100 |
| DPK10 | 0.116 | 0.116 |
| DPK11 | 0.094 | 0.094 |
| DPK12 | 0.124 | 0.124 |
| DPK13 | 0.122 | 0.121 |
| DPK14 | 0.118 | 0.119 |
| DPK15 | 0.113 | 0.113 |
| DPK16 | 0.102 | 0.102 |
| MEDIAN | 0.100 | 0.100 |
