# Supplementary material for: Rates of CTL Killing in Persistent Viral Infection In Vivo
Source: PLoS Comput Biol. 2014 Apr 3;10(4):e1003534. doi: 10.1371/journal.pcbi.1003534 (PMC3974637; doi:10.1371/journal.pcbi.1003534)
Supplement: Table S9 — Average number of divisions that an average CFSE- or PKH26-positive cell would have to undergo to become label-negative. (DOCX) [file pcbi.1003534.s012.docx]

| **animal ID** | **CFSE B cells** | **PKH26 B cells** |
| --- | --- | --- |
| **BLV1** | 5.88 | 6.10 |
| **BLV2** | 6.80 | 6.47 |
| **BLV3** | 6.63 | 6.66 |
| **BLV4** | 6.24 | 5.81 |
| **BLV5** | 6.23 | 5.76 |
| **BLV6** | 6.53 | 6.15 |
| **CsA1** | 6.29 | 6.18 |
| **CsA2** | 6.11 | 5.87 |
| **CsA3** | 6.29 | 6.13 |
| **NI1** | 5.76 | 5.83 |
| **NI2** | 6.12 | 6.05 |
| **NI3** | 6.49 | 6.27 |
| **mean** | **6.28** | **6.11** |
