# Supplementary material for: Rates of CTL Killing in Persistent Viral Infection In Vivo
Source: PLoS Comput Biol. 2014 Apr 3;10(4):e1003534. doi: 10.1371/journal.pcbi.1003534 (PMC3974637; doi:10.1371/journal.pcbi.1003534)
Supplement: Text S1 — Other estimates of CTL killing from the literature. (DOCX) [file pcbi.1003534.s013.docx]

**Other Estimates of CTL Killing from the Literature**

The rate of CTL killing has been estimated in multiple studies for three virus infections namely LCMV, Polyoma virus and HIV-1/SIV. The rates were expressed in different units in the different studies making comparisons difficult. Here we convert all rates to the same units. Killing can be expressed either per capita CTL (where CTL can be measured as a frequency or an absolute count) or for the overall CTL response (i.e. the death rate of an infected cell attributable to CTL); additionally killing can be measured for a single CTL response (i.e. T cell clone or clones specific for a single epitope) or for the total CTL response (i.e. all T cells specific for a particular virus). Here we convert all rates to killing by the overall total CTL response, where this is not possible we convert to killing by the overall single CTL response. Target cell lifespan is 1/killing rate, target cell half life is ln(2)/killing rate. Results are summarised in Table S1.

**Acute LCMV infection**

Barchet et al were the first to quantify the disappearance of LCMV-infected cells [[1](#_ENREF_1)]. However, their estimate does not solely include disappearance due to CD8 killing and can thus not be converted to the killing rate of the overall total CTL response. The data of Barber et al [[2](#_ENREF_2)] in which the fate of peptide-pulsed splenocytes was tracked in mice acutely infected with LCMV has been analysed in 3 studies.

**Regoes et al [**[**3**](#_ENREF_3)**]**

Regoes et al quantified CTL killing per capita (where number of CTL were measured as a frequency) and for a single response. To convert to overall killing due to a single response it is necessary to multiply by the frequency of CTL. In acute infection, Regoes et al report a killing rate of 1.33 per minute per CTL for CTL specific for the NP396 peptide and 0.70 per minute per CTL for CTL specific for the GP276 peptide. The supplementary material provides the frequency of CD8+ T cells specific for each epitope and the frequency of CD8+ T cell in the spleen yielding the frequency of specific CD8+ T cells in the spleen as 0.066 for NP396-specific CD8+ T cells and 0.021 for GP276-specific CD8+ T cells averaged over all time points during the killing assay. The overall killing rate for the NP396-specific response is thus 126.4d^-1^ and for the GP276-specific response it is 21.2d^-1^.

**Yates et al [**[**4**](#_ENREF_4)**]**

Yates et al extended the modelling work of Regoes et al and expressed killing in the same units. They report a killing rate of 3.71 per minute per CTL for CTL specific for the NP396 peptide and 2.19 per minute per CTL for CTL specific for the GP276 peptide. Converting to the overall killing rate as above yields 352.6d^-1^ for the NP396-specific response and 66.2d^-1^ for the GP276-specific response.

**Ganusov & de Boer [**[**5**](#_ENREF_5)**]**

Ganusov & de Boer also model the Barber data but take a different modelling approach. They calculate the overall killing rate for a single response and express it as a half-life of targets. This is trivially converted to an overall killing rate by taking the reciprocal and multiplying by ln(2). They report half-lives of 2 minutes and 14 minutes for splenocytes pulsed with the NP396 and GP276 peptides respectively. Yielding overall killing rates of 499.1d^-1^ for the NP396-specific response and 71.3d^-1^ for the GP-276-specific response.

**Chronic LCMV infection**

**Graw et al [**[**6**](#_ENREF_6)**]**

Graw et al modelled the loss of splenocytes pulsed with NP396 and GP33 peptides in mice persistently infected with LCMV. The median half-life of targets in the individual mice was estimated as 328.9 minutes and 23.63 minutes for NP396- and GP33-pulsed targets respectively. Converting to the overall killing rate of a single CTL response as above yields a killing rate of 3.0d^-1^ and 42.2d^-1^ respectively. In Figure 5 in the main text we represent killing attributable to the total CTL response. To convert killing attributable to a single CTL response to killing attributable to a total response it is necessary to scale up. We assume that CTL responses add up in a linear fashion. Studies suggest that there are 10 to 28 LCMV-specific CTL responses [[7](#_ENREF_7),[8](#_ENREF_8)]. These are unlikely to all be effective so we take the conservative estimate of 10. We thus estimate the total CTL killing rate as 5x(3.0+42.2)=226d^-1^.

**Polyoma virus infection**

**Ganusov et al [**[**9**](#_ENREF_9)**]**

Ganusov et al calculated the per capita overall killing rate for a single response against Polyoma virus in mice. They found an overall killing rate for MT389 peptide-pulsed splenocytes by the specific CTL response of 4.16min^-1^ in acute infection and 1.90min^-1^ in chronic infection. The average frequency of MT389-specific CTL in the spleen was found to be 1.13% in the acute phase and 0.79% in the chronic phase. Using this information to convert to the overall killing rate as explained for LCMV infection yields 67.7d^-1^ in the acute phase and 21.6d^-1^ in the chronic phase.

**Chronic HIV-1 infection**

**Wick et al [**[**10**](#_ENREF_10)**]**

Wick et al analysed a clinical procedure [[11](#_ENREF_11)] in which CD8+ T cells were isolated from 3 HIV-1-infected patients, expanded in vitro with HIV-1 peptides and then reinfused. They estimated that HIV-1-specific CD8+ T cells killed productively infected cells at a rate of 0.14 μl cell^-1^d^-1^ in patient 1, 0.17 μl cell^-1^d^-1^ in patient 2 and 0.076 μl cell^-1^d^-1^ in patient 3. To express this as overall killing due to the total CTL response these numbers need to be multiplied by the number of HIV-specific CD8+ T cells per μl of plasma. Reference [10] gives a median CD8 count of 500 cells μl^-1^ and an average frequency of HIV-1-specific CD8+ T cells of 10% i.e. a count of 50 specific CD8 μl^-1^. Reference [11] gives a median CD8 count of 860 cells μl^-1^ and an average frequency of HIV-1-specific CD8+ T cells of 7.5% i.e. a count of 65 specific CD8 μl^-1^. Averaging across these two estimates yields a count of 57.5 specific CD8 μl^-1^. So Wick’s estimates are equivalent to a killing rate attributable to the overall total response of 8.1d^-1^ in patient 1, 9.8d^-1^ in patient 2 and 4.4d^-1^ in patient 3.

**Asquith et al [**[**12**](#_ENREF_12)**]**

We estimated the rate of CD8+ T cell killing from the antiviral selection pressure which is manifest as HIV-1 escape from the CD8+ T cell response in HLA-matched hosts (i.e. hosts with the HLA class I molecule to present the epitope) and reversion in HLA-mismatched hosts (lacking the presenting HLA molecule). In 21 cases in late primary/ chronic HIV-1 infection the median rate of escape was 0.01d^-1^, adding this to the median rate of reversion (0.005d^-1^) yields the reported median overall killing rate of 0.02d^-1^ for a single response or 0.2d^-1^ for the total response [[12](#_ENREF_12)]. In figure 5 the distribution is estimated by random resampling with replacement from the escape rates and random resampling with replacement from the reversion rates and then summing the estimates to obtain the overall rate of killing attributable to a single CTL response and then extrapolating to the total CTL response as in [[12](#_ENREF_12)].

**Acute HIV-1**

**Goonetilleke et al [**[**13**](#_ENREF_13)**]**

Goonetilleke et al calculate CD8+ T cell antiviral pressure in very early acute HIV-1 infection following the method of Asquith et al [[12](#_ENREF_12)]. They report a median escape rate from a single CTL response of 0.14d^-1^. They do not estimate rate of reversions though these are typically at least an order of magnitude slower than 0.14d^-1^ [[12](#_ENREF_12)] and thus the median rate of killing by a single CTL response can be estimated as 0.14d^-1^. It is not known how many CTL responses are of sufficient strength to drive escape in acute infection so extrapolating from the killing attributable to a single response to the killing attributable to the total response is difficult. If we follow the approach of Asquith et al and apply it to acute infection we find that the rate of killing attributable to the total response is 0.14x5+0.007x14=0.80d^-1^. However, this is may be an overestimate as there are fewer CTL responses in acute than in chronic HIV-1 infection.

**Acute SIV-1 infection**

**Mandl et al [**[**14**](#_ENREF_14)**]**

Mandl et al estimated the killing rate of Tat SL8-specific CTLs in acute SIV-infection using the method described by Asquith et al [[12](#_ENREF_12)]. They report a per capita estimate of 0.016 μl cell^-1^d^-1^ for a group of four SIV-infected rhesus macaques. To convert this estimate to overall killing rate per day the number needs to be multiplied by the number of HIV-specific CD8+ T cells per μl of plasma. We estimate the average number of specific CTLs of the four animals at the peak of CTL escape is ~ 45 cells per μl [[14](#_ENREF_14)], resulting in a killing rate of 0.7d^-1^ for the overall single CTL response.

**Petravic et al [**[**15**](#_ENREF_15)**]**

Petravic et al estimated the rate of CTL killing from observed escape and reversion rates from the KP9-specific CD8+ T cell response in SHIV-infection. Reversion rate is calculated from the absolute fitness costs of the escape mutation at any point in time and the number of target cells available. They estimated a killing rate for the overall single CTL response of 0.05-0.61d^-1^.

**Chronic SIV-1 infection**

**Asquith et al [**[**16**](#_ENREF_16)**]**

Using the approach of [[12](#_ENREF_12)] we estimated the rate of escape from the natural (i.e. not vaccine induced) CTL response to be 0.02 d^-1^ in chronic and acute infection and the rate of reversion to be 0.1d^-1^, yielding a median rate of killing of the overall single CTL response of 0.12d^-1^.

**Elemans et al [**[**17**](#_ENREF_17)**]**

We quantified the contribution of the total CTL response to productively infected cell death. The average contribution of the total CTL response in 11 animals was 41.8%. The death rate of productively infected cell has previously been estimated to be 1d^-1^ [[18](#_ENREF_18)], resulting in an overall total CTL killing rate of 0.4d^-1^.

**Elemans et al [**[**19**](#_ENREF_19)**]**

Based on the assumption that CD8+ T cells control viraemia by a purely lytic mechanism and target cell numbers are constant, then the rate of increase of viral load following CD8+ T cell depletion is equal to the rate of lysis of productively infected cells we estimated an overall total killing rate of 0.3d^-1^ in SIV infection.

References

1. Barchet W, Oehen S, Klenerman P, Wodarz D, Bocharov G, et al. (2000) Direct quantitation of rapid elimination of viral antigen-positive lymphocytes by antiviral CD8+ T cells in vivo. European Journal of Immunology 30: 1356-1363.

2. Barber DL, Wherry EJ, Ahmed R (2003) Cutting edge: Rapid in vivo killing by memory CD8 T cells. Journal of Immunology 171: 27-31.

3. Regoes RR, Barber DL, Ahmed R, Antia R (2007) Estimation of the rate of killing by cytotoxic T lymphocytes in vivo. Proc Natl Acad Sci U S A 104: 1599-1603.

4. Yates A, Graw F, Barber DL, Ahmed R, Regoes RR, et al. (2007) Revisiting estimates of CTL killing rates in vivo. PLoS ONE 2: e1301.

5. Ganusov VV, De Boer RJ (2008) Estimating in vivo death rates of targets due to CD8 T-cell-mediated killing. J Virol 82: 11749-11757.

6. Graw F, Richter K, Oxenius A, Regoes RR (2011) Comparison of cytotoxic T lymphocyte efficacy in acute and persistent lymphocytic choriomeningitis virus infection. Proc Biol Sci 278: 3395-3402.

7. Masopust D, Murali-Krishna K, Ahmed R (2007) Quantitating the magnitude of the lymphocytic choriomeningitis virus-specific CD8 T-cell response: It is even bigger than we thought. Journal of Virology 81: 2002-2011.

8. Kotturi MF, Peters B, Buendia-Laysa F, Jr., Sidney J, Oseroff C, et al. (2007) The CD8(+) T-cell response to lymphocytic choriomeningitis virus involves the L antigen: Uncovering new tricks for an old virus. Journal of Virology 81: 4928-4940.

9. Ganusov VV, Lukacher AE, Byers AM (2010) Persistence of viral infection despite similar killing efficacy of antiviral CD8(+) T cells during acute and chronic phases of infection. Virology 405: 193-200.

10. Wick WD, Yang OO, Corey L, Self SG (2005) How many human immunodeficiency virus type 1-infected target cells can a cytotoxic T-lymphocyte kill? J Virol 79: 13579-13586.

11. Brodie SJ, Lewinsohn DA, Patterson BK, Jiyamapa D, Krieger J, et al. (1999) In vivo migration and function of transferred HIV-1-specific cytotoxic T cells. Nat Med 5: 34-41.

12. Asquith B, Edwards CT, Lipsitch M, McLean AR (2006) Inefficient cytotoxic T lymphocyte-mediated killing of HIV-1-infected cells in vivo. PLoS Biol 4: e90.

13. Goonetilleke N, Liu MK, Salazar-Gonzalez JF, Ferrari G, Giorgi E, et al. (2009) The first T cell response to transmitted/founder virus contributes to the control of acute viremia in HIV-1 infection. J Exp Med 206: 1253-1272.

14. Mandl JN, Regoes RR, Garber DA, Feinberg MB (2007) Estimating the effectiveness of simian immunodeficiency virus-specific CD8(+) T cells from the dynamics of viral immune escape. Journal of Virology 81: 11982-11991.

15. Petravic J, Loh L, Kent SJ, Davenport MP (2008) CD4(+) target cell availability determines the dynamics of immune escape and reversion in vivo. Journal of Virology 82: 4091-4101.

16. Asquith B, McLean AR (2007) In vivo CD8(+) T cell control of immunodeficiency virus infection in humans and macaques. Proceedings of the National Academy of Sciences of the United States of America 104: 6365-6370.

17. Elemans M, Thiebaut R, Kaur A, Asquith B (2011) Quantification of the Relative Importance of CTL, B Cell, NK Cell, and Target Cell Limitation in the Control of Primary SIV-Infection. PLoS Computational Biology 7: e1001103.

18. Markowitz M, Louie M, Hurley A, Sun E, Di Mascio M (2003) A novel antiviral intervention results in more accurate assessment of human immunodeficiency virus type 1 replication dynamics and T-Cell decay in vivo. Journal of Virology 77: 5037-5038.

19. Elemans M, al Basatena N-KS, Klatt NR, Gkekas C, Silvestri G, et al. (2011) Why Don't CD8+T Cells Reduce the Lifespan of SIV-Infected Cells In Vivo? PLoS Computational Biology 7.
