# Supplementary material for: Rates of CTL Killing in Persistent Viral Infection In Vivo
Source: PLoS Comput Biol. 2014 Apr 3;10(4):e1003534. doi: 10.1371/journal.pcbi.1003534 (PMC3974637; doi:10.1371/journal.pcbi.1003534)
Supplement: Text S2 — Additional models. (DOCX) [file pcbi.1003534.s014.docx]

**Additional Models**

To test the robustness of our estimates or CTL killing to model assumptions we considered a number of alternative models. In addition to the two models described in the main text, one in which we estimated the fraction of ag^+^ CFSE^+^ infected cells as a free parameter and one in which we set the fraction of infected cells at the start of the experiment equal to the proviral load (rather than calculating it from the proviral load), the tested three further models. Firstly, we tested a model in which we included a damaged population that has a reduced lifespan due to the treatment and therefore dies quickly after reinjection. We found that this model gives a similar fit to the data but a higher AICc due to an extra parameter. The estimates of the killing rate of the BLV-infected untreated animals are in the same order of magnitude as the original model (median 1.6 d^-1^). If we fit a model in which we assume the same proliferation rate for ag^+^ and ag^─^ infected cells we again find killing estimates in the same order of magnitude (median 1.1 d^-1^) suggesting simplification of the model has only a small impact on killing rate estimates. Finally, we tested a model in which uninfected cells can become infected and become ag^─^ infected cells. Infection rate is dependent on the size of the ag^+^ population. This model gave similar or slightly better fits to the data, but a higher AIC_c_ due to an extra parameter. Again, estimates of CTL killing rate were in the same order of magnitude as in the original model (median 2.8 d^-1^).

We conclude that different models can fit the data but the killing estimates do not change significantly. We presented the best supported model that was consistent with the known biology.
