# Supplementary material for: Rates of CTL Killing in Persistent Viral Infection In Vivo
Source: PLoS Comput Biol. 2014 Apr 3;10(4):e1003534. doi: 10.1371/journal.pcbi.1003534 (PMC3974637; doi:10.1371/journal.pcbi.1003534)
Supplement: Text S3 — Circulation model. (DOCX) [file pcbi.1003534.s015.docx]

**Circulation model**

To include circulation in the model we adjusted the model so each population has a blood and lymphoid component, denoted by B and L subscripts respectively. Killing, proliferation, death and up-regulation of Tax are assumed to take place in the lymphoid compartment.

$\dot{S_{B0}}=rS_{L0}-d_{B}S_{B0}-hS_{B0}$ (1a)

$\dot{S_{L0}}=hS_{B0}-\left( u+d+p_{s}+r \right)S_{L0}$ (1b)

$\dot{S_{Bi}}=rS_{Li}-d_{B}S_{Bi}-hS_{Bi}$ (2a)

$\dot{S_{Li}}=hS_{Bi}+2p_{s}S_{Li-1}-\left( u+d+p_{s}+r \right)S_{Li}$ (2b)

$\dot{S_{Bn}}=rS_{Ln}-d_{B}S_{Bn}-hS_{Bn}$ (3a)

$\dot{S_{Ln}}=hS_{Bn}+2p_{s}S_{Ln-1}+\left( p_{s}-u-d-r \right)S_{Ln}$ (3b)

$\dot{T_{B0}}=rT_{L0}-d_{B}T_{B0}-hT_{B0}$ (4a)

$\dot{T_{L0}}=hT_{B0}+uS_{L0}-\left( k+d+p_{b}+r \right)T_{L0}$ (4b)

$\dot{T_{Bi}}=rT_{Li}-d_{B}T_{Bi}-hT_{Bi}$ (5a)

$\dot{T_{Li}}=hT_{Bi}+uS_{Li}+2p_{b}T_{Li-1}-\left( k+d+p_{b}+r \right)T_{Li}$ (5b)

$\dot{T_{Bn}}=rT_{Ln}-d_{B}T_{Bn}-hT_{Bn}$ (6a)

$\dot{T_{Ln}}=hT_{Bn}+uS_{Ln}+2p_{b}T_{Ln-1}+\left( p_{b}-k-d-r \right)T_{Ln}$ (6b)

$\dot{H_{B0}}=rH_{L0}-d_{B}H_{B0}-hH_{B0}$ (7a)

$\dot{H_{L0}}=hH_{B0}-\left( d+p_{s}+r \right)H_{L0}$ (7b)

$\dot{H_{Bi}}=rH_{Li}-d_{B}H_{Bi}-hH_{Bi}$ (8a)

$\dot{H_{Li}}=hH_{Bi}+2p_{s}H_{Li-1}-\left( d+p_{s}+r \right)H_{Li}$ (8b)

$\dot{H_{Bn}}=rH_{Ln}-d_{B}H_{Bn}-hH_{Bn}$ (9a)

$\dot{H_{Ln}}=hH_{Bn}+2p_{s}H_{Ln-1}+\left( p_{s}-d-r \right)H_{Ln}$ (9b)

Where h is the rate at which cells leave the blood, r the rate at which they leave the lymphoid compartment and d_B_ the death rate in the blood (all d^-1^) . The blood and lymphoid compartments are in steady state we defined r=h/s, where s is a scaling factor between the size of the blood lymphocyte population and lymphoid lymphocyte compartment. Assuming infection rate is negligibly small we can set $\dot{H}=0$, from which follows $d=p_{s}$, and $\left( \dot{T}+\dot{S} \right)=0$, allowing to set $p_{s}=p_{b}-k$.

We fixed the rate at which cells leave the blood h over a range from 1d^-1^ to 120d^-1^ and fit the other parameters. Estimated killing rates (Table S7) were more similar between the animals but in the same order of magnitude as in the original model. We did not find a clear effect of the rate at which cells leave the blood on the estimated killing rate. We found a median killing rate of 1.64 d^-1^ in the circulation model compared to 1.98 d^-1^ in the original model.

The killing estimate is largely determined by the difference in the decline of CFSE-labelled cells and PKH-labelled cells. If PKH and CFSE labelled cells migrate at similar rates then a fast or slow migration rate would affect the dynamics of both populations and have little impact on killing rates. However, if CFSE-labelled cells migrated more rapidly to lymphoid tissues and/or were preferentially retained there then the faster decline in the percentage of CFSE labelled cells compared to PKH26-labelled cells would (at least) partly be explained by this difference in migration rate and would lead to lower killing estimates.

Compared with the original model we found a higher initial fraction of ag^+^ cells in the PKH26-labelled population and a higher up-regulation rate, u; proliferation rate p_b_ was more similar between the animals but in the same order of magnitude.

For scaling between blood and lymphoid compartment we found values between 0.69 and 7.63 over all model fits and a median of 2.76. In the literature a blood/lymph ratio for the concentration of circulating lymphocytes of 3.5 has been reported [[1](#_ENREF_1)]. The ratio in absolute numbers is in a similar order of magnitude, assuming the blood volume is about equal to the lymph volume [[2](#_ENREF_2)].

References

1. Andrade WN, Johnston MG, Hay JB (1998) The relationship of blood lymphocytes to the recirculating lymphocyte pool. Blood 91: 1653-1661.

2. Manery JF (1954) Water and electrolyte metabolism. Physiological reviews 34: 334-417.
